# How does the subjective well-being of Australian adults with a congenital corpus callosum disorder compare with that of the general Australian population?

**DOI:** 10.1007/s11136-024-03741-w

**Published:** 2024-07-24

**Authors:** Maree Maxfield, Keith McVilly, Alexandra Devine, Christian Davey, Helen Jordan

**Affiliations:** 1 Melbourne School of Population and Global Health, University of Melbourne, Victoria, Australia; 2 School of Social and Political Sciences, University of Melbourne, Victoria, Australia; 3 School of Mathematics and Statistics, University of Melbourne, Victoria, Australia

**Keywords:** Corpus callosum, Personal wellbeing, Adults, Quality of life, PWI, Brain

## Abstract

**Purpose:**

Very little is known about the subjective well-being (SWB) of adults with a congenital corpus callosum disorder (CCD), the extent to which they feel satisfied with their lives, and what might be helpful in improving their SWB and quality of life. This study measured SWB among Australian adults with a CCD and compared the results with normative data for the wider Australian adult population.

**Methods:**

Online surveys were completed independently by 53 Australian adults with a CCD. Data included demographic profiles and answers to questions about satisfaction with life, employing the Personal Wellbeing Index (PWI) and one open ended question. Domains measured included life as a whole, standard of living, health, achieving in life, personal relationships, safety, community connectedness and future security. The PWI results were statistically analysed and means compared with Australian normative data. The qualitative data were analysed using deductive thematic analysis.

**Results:**

Australian adults with a CCD responded with ratings significantly below what might be expected of the adult Australian population in all domains except for standard of living and safety. Quantitative analysis results were supported by qualitative thematic analysis, expressing particular challenges and barriers to feeling satisfaction with life as a whole, personal relationships, achieving in life, health and future security.

**Conclusion:**

Evidence from the PWI and accompanying qualitative responses indicate that SWB of Australian adults with CCD is significantly reduced compared with the general population. Further research is needed to examine the lived experience and explore solutions for support of this community.

## Abstract (plain language summary)

Very little is known about the quality of life and personal well-being of Australian adults who have a corpus callosum disorder (CCD), a rare brain condition with complex impacts ranging from mild to severe. People with a CCD are born missing part or all of their corpus callosum, the connecting body between the two halves of the brain. This study asked adults with a CCD to rate their own personal wellbeing in an online questionnaire and the results were compared with those of the general Australian population. Participants were also asked to describe how a CCD affected their lives in their own words. We found that adults with a CCD have significantly lower personal wellbeing than the general Australian population. The most concerning issues were with relationships, life achievements, mental health and not having a secure future. The results tell us that we need to explore personal wellbeing in more depth using research methods in which adults with a CCD can tell us more about the impacts of a CCD on their lives. We need to ask how they think the quality of their lives could be improved and what supports would help to achieve that.

## Introduction

Of the 38 countries in the Organization for Economic Co-operation and Development (OECD), people living in Australia enjoy some of the highest standards of living and satisfaction with life [1]. However, compared to the general population, Australian adults with rare diseases are at risk of poorer health, reduced economic and social participation, and as a consequence poorer quality of life and well-being [2–9]. Research and our understanding of the quality of life of Australians with rare diseases remain sparse, including among adults with a congenital corpus callosum disorder (CCD) [7, 10, 11].

Composed of more than 200 million nerve fibres, the corpus callosum is the major connecting body, enabling cognitive, motor, and sensory communication between the two hemispheres of the brain [12]. A CCD presents in 1:4000 live births, occurring when the corpus callosum forms atypically in utero and is partially or completely absent, or misshapen at birth [12, 13]. Prior to advances in prenatal imaging enabling diagnosis in utero, estimations of prevalence in adults had relied upon symptomatic individuals presenting to clinics, with some adults typically being undiagnosed or misdiagnosed [12]. Cause and presentation of CCDs are highly heterogeneous, with diverse cognitive, physical, and psychologically disabling impacts ranging from mild to severe in addition to associated diagnoses including autism, intellectual disability, epilepsy, and cerebral palsy. Psychiatric disorders may also be present [14–16].

Adults with CCD are typically geographically and socially isolated from peers. Barnby et al. (2022) report that increased credulity and persuadability potentially expose them to ‘social trickery’ [17 p. 260] and manipulation by others, negatively impacting on personal choice, control, and independence in their lives [17, 18]. They experience social inequity through barriers to education, employment, and relationships, with reduced access to services and supports [19–21]. Knowledge around this heterogeneous, rare brain disorder is limited. Unless health and disability professionals have accurate knowledge of rare disorders, individual support is often inadequate, inconsistent or unintentionally misdirected, perpetuating stigma, and adversely affecting quality of life and well-being [3, 17, 19, 20, 22]. Although there is a growing body of biomedical literature reporting on the cognitive, physical, and psychological impacts of a CCD, there is a paucity of literature examining the quality of life and well-being of adults with a CCD, of which understanding is critical to the development and delivery of effective services and other supports [15].

### Quality of life and well-being

The terms ‘quality of life’ and ‘well-being’ (wellbeing) whilst commonly used interchangeably, are convergent but not the same [23–25]. Quality of life refers to a cognitive appraisal of an individual’s situation, whereas well-being is an emotional response to how an individual experiences their life [26]. The World Health Organization (WHO) defines quality of life as “an individual’s perception of their position in life in the context of the culture and value systems in which they live, and in relation to their goals, expectations, standards and concerns” [27 p. 3]. Social status, social participation and networks, life events, and relationships with family and friends all have positive or negative correlations with quality of life [28]. Well-being is dependent on cognitive, physical, and social influences, in addition to an individual’s experience of ‘life as a whole’ and the functional environment in which they live, and the extent to which that environment is supportive of their needs and aspirations [26, 28–30]. The Australian Centre on Quality of Life (ACQOL) website identifies dimensions of objectivity and subjectivity where “each of these two dimensions comprises several domains which, together, define the total construct. Objective domains are measured through culturally relevant indices of objective well-being. Subjective domains are measured through questions of satisfaction” [31].

### Measuring well-being

To complement economic indicators to measure a whole society’s well-being, there is growing recognition that holistic quality of life and the subjective well-being (SWB) of individuals are important constructs to consider when measuring the well-being of populations [1, 32–36]. SWB is about how an individual experiences and appraises their own life and can only be measured through directly asking questions of individuals whose life is being considered [1, 30, 37]. Here it should be noted that proxy data relating to SWB risk ‘inherent biases’ and are not regarded as valid. SWB focuses on an individual’s personal emotional response to their circumstances [30]. Yet, for people with disability, measures of well-being have often been limited to reliance on objective indicators and proxy informed data [30, 33, 38, 39].

SWB in Western nations is typically measured at an average level of 75% points (pp) on the Personal Wellbeing Index (PWI) scale of 0 -100, with temporary fluctuations occurring through a combination of incidental and enduring emotional experiences affecting mood [28, 33, 40]. In human psychology, maintaining homeostatic SWB levels is analogous to maintaining stable biological levels in physical function, such as blood pressure or core body temperature [40, 41]. Biological homeostatic systems fluctuate with physiological challenges, whereas SWB homeostasis is challenged by positive or negative emotional experiences. Fluctuations typically return to a homeostatic point of equilibrium [42]. For SWB, recovery from emotional challenges and maintenance of equilibrium is dependent on the strength of the challenges together with the personal resources available to the individual. The social environment plays a critical role in the ‘life stress’ process involving psychological distress [43]. Weak emotional challenges have little impact on homeostatic stability. Strong and persistent emotional challenges such as poverty and chronic anxiety present stressors that can overwhelm homeostatic recovery, causing chronic homeostatic failure, observed in ratings of SWB that fall significantly below the population norm. [18, 33, 42]. Key resources in the social environment known to defend against homeostatic failure or assist with recovery are relationships, financial control, and achieving in life, described by Cummins as the ‘Golden Triangle’ [40, 43, 44]. Many of these resources can be deficient in the lives of those with a CCD.

### The current study

Very little is known about the SWB of adults with a congenital CCD, the extent to which they feel satisfied with their lives and what might be helpful in improving their SWB. The purpose of our study was therefore to address this gap by employing the self-reporting PWI to measure SWB among Australian adults with a CCD, and compare the results with those of the wider Australian adult population to inform future research and service provision [30, 38, 45]. This study addressed the questions; (1) *Using the PWI, how do Australian adults with CCD rate their subjective well-being (SWB);* and (2) *How does the SWB of Australian adults with a CCD compare with that of the general Australian population?*

## Method

This study was conducted via an anonymous on-line survey. The on-line methodology was adopted to maximise participation by a relatively small but geographically distributed population, and to accommodate public health regulations associated with the COVID-19 pandemic. It was descriptive in nature, employing the PWI [30] to collect quantitative responses to standardized questions, alongside one qualitative, open-ended question, inviting participants to provide further comments on their experiences.

### Recruitment & data collection

Participants were recruited via websites, social media, newsletters, support groups, medical and allied health practices, and research organisations. Potential participants were eligible if they were Australian, aged 18 years or older, diagnosed with a congenital CCD, and able to give informed consent and independently complete the online survey. Diagnosis of a congenital CCD included complete, partial, hypoplasia, and dysgenesis of the corpus callosum, collectively referred to as corpus callosum disorders (CCD).

Data were collected from December 2021 to June 2023. Participants followed a link to Qualtrics online, where hyperlinks were provided to standard and accessible, written and oral versions of the Plain Language Statement. As part of the online, informed consent process, participants were asked to agree to complete the survey on their own (i.e. noting independent completion is a key component of measuring SWB). Alongside the PWI items, participants were asked to provide basic demographic data (e.g. age, type of CCD) and given the option to add comments as a free text response to the question, “*Is there anything else you would like to tell us about how a corpus callosum disorder affects your life*?” At the completion of the online survey, participants were eligible to register on a separate online list to receive a $20 gift voucher in appreciation.

### Measures

The PWI, noted by the OECD and WHO as a preferred scale, measures self-reported SWB among adults [33, 36, 46, 47]. The PWI has also been used to measure SWB for children and young people, and for people with physical and cognitive disability [4, 48, 49].

**Fig. 1:**
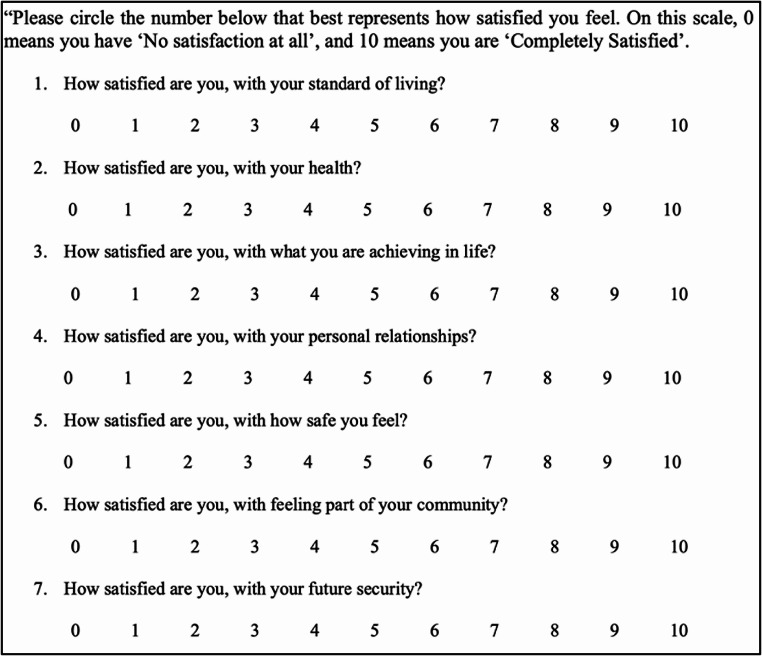
PWI questions of satisfaction for seven subjective well-being domains [50]

The PWI has two main components. It begins with the simplest measure of Global Life Satisfaction, asking the question, “*How satisfied are you with your life as a whole?*” [30]. It then deconstructs Life as a Whole, asking the questions shown in Fig. 1, to measure satisfaction across seven established SWB domains: *(1) Standard of Living, (2) Health, (3) Achieving in Life, (4) Personal Relationships, (5) Safety, (6) Community Connectedness, and (7) Future Security* [30, 50]. Domain scores can be analysed individually, as well as averaged to calculate the PWI score. In the current study, respondents were asked to rate their satisfaction against the Life as a Whole and seven PWI satisfaction questions using an 11-point, unipolar Likert scale where 0 indicates ‘*No satisfaction at all’* and 10 indicates ‘*Completely satisfied*’ [50] (Fig. 1). Participants were given the opportunity to answer the single, additional open text response question collecting qualitative data.

The broad, semi abstract characteristics of the questions in the PWI Domains meet accepted SWB criteria required to produce a robust instrument. The PWI has a Cronbach’s alpha of 0.80–0.90 [45], and as a measure of SWB in Australia, has demonstrated reliability and stability over decades [40, 50, 51].

### Data analysis: Quantitative

The quantitative data set consisted of the responses to the ‘Life as a Whole’ question, 7 PWI key Domain questions, and a PWI score. Prior to analysis, the Life as a Whole and 7 SWB domains on the Likert scales of 0 to 10, were converted to a percentage of scale maximum (%SM) score by shifting the decimal point one space to the right, as determined by the PWI Manual [50]. The PWI score was defined as the mean of the seven key SWB Domain percentage scores (but not including Life as a Whole).

To check the internal consistency of the PWI completed by study participants, Cronbach’s alpha was computed using the function from the Ludecke et al. performance package [52]. For each variable, means were calculated from the data and estimates of the Standard Error (SE) and a 95% Confidence Interval (CI) for each variable computed using boot R packages [53, 54]. Using a Welch-Satterthwaite two sample t-test, results of the participants’ self-reported PWI survey were then compared with normative ranges of the general Australian adult population, calculated from aggregated survey mean scores from 2002 to 2021 [46, Table 4.1]. The construction and large size of these surveys renders negligible any uncertainty concerning their results.

**Data analysis: Qualitative**

Statistical results were supplemented with qualitative thematic analysis of the open-ended text response collected at the end of the survey. Informed by Braun and Clarke [55], qualitative analysis was conducted manually, employing deductive thematic analysis of the participant responses to identify “patterns of shared meaning, underpinned by a central organising concept” [55 p 230] around participants’ perspectives. The respondents’ comments were tabulated and coded to the 8 apriori domains of the PWI instrument. This process included the application of repeated reading of comments and where needed, consensus coding in collaboration with multiple coders (the authors).

## Results

Sixty-eight participants completed the online survey. Fifteen responses were excluded as they were incomplete, duplicate submissions, or from participants outside Australia. The final sample included 53 participants (31 female), aged 18 to 78 years (mean 39.6 years). For details of the demographic profiles see Table 1.

**Table 1:** Demographic profiles of the study sample of adults with a corpus callosum disorder (*n* = 53)

Demographic variables	*n*	%
**Age in years** (m = 39.6, SD = 14.9)		
18–24	9	16.9
25–34	13	24.5
35–44	11	20.8
45–54	12	22.6
55–64	5	9.4
65 or over	3	5.7
**Gender**		
Female	31	58.5
Male	22	41.5
Other	0	0
**CCD diagnosis**		
Complete ACC	32	60.3
Partial ACC	18	34.0
Hypoplasia	3	5.7
Hyperplasia	0	0.0
**Education**		
Year 10	9	16.9
Year 12	17	32.1
Technical and Further Education (TAFE)	13	24.5
University Undergraduate	11	20.8
University Postgraduate	3	5.7
**Employment**		
Currently in full time paid employment	11	20.8
Currently in part time paid employment	14	26.4
Not currently in paid employment (have in past)	16	30.2
Worked as a volunteer	8	15.1
Never worked In paid employment or as a volunteer	4	7.5
**Current living situation**		
With partner or spouse	21	39.6
With other family	21	39.6
With friends	3	5.7
Alone	7	13.2
Other	1	1.9
**Income Source**		
Disability Support Pension	19	35.9
Other Centrelink (Government support payment)	5	9.4
Employment	20	37.7
Other	7	13.2
Prefer not to answer	2	3.8
**National Disability Insurance Scheme Participant** ^(a)^		
Applied & have a plan	22	41.5
Applied and refused	5	9.4
No, but planning to	4	7.5
No, and not planning to	16	30.2
Other	6	11.3

^(a)^An Australian government scheme that provides funding to eligible Australians with disability to support greater independence, access to new skills, jobs or volunteering, and an improved quality of life [56]

### PWI quantitative results

Given that the sample mean, sample standard deviation, and number of samples have been reported for each group, the statistical significance of each outcome was assessed using a two-sample *t*-test, with the approximate degrees of freedom computed via the Welch-Satterthwaite equation. In accordance with the PWI manual [45, 46] the participant sample means were compared with normative data for Australian adults, comprising means of 36 population studies spanning 20 years (2002–2021).

Mean scores for Life as a Whole, the PWI score, and the seven SWB domains of the deconstructed PWI score are shown in Table 2. For Life as a Whole, survey participants reported a mean score of 66.6 with scores ranging from approximately 61 to 72 (95%CI). A similar range of approximately 61 to 70 (95%CI) was reported for the PWI score with a mean of 65.6. Across our 53 survey participants, Cronbach’s alpha for the seven items used to calculate the PWI was 0.81, indicating high internal consistency, comparable with that of the Australian population normative data (0.80–0.90) [45, 46].

Participants’ results were compared with normative data for Australian adults, based on an analysis of 36 population studies (*N* = 65,722), spanning 20 years (2002–2021). The comparative sample reported a mean score for Life as a Whole of 77.4 and a mean PWI Score of 75.5. Statistical significance for each measured item was assessed using a two-sample *t*-test. As shown in Table 2, CCD participants reported SWB ratings for Life as a Whole significantly below that of the Australian normative sample; *t*(52) = 4.15, *p* < .001. Similarly, CCD participants reported ratings for the PWI score significantly below that of the Australian normative sample; *t*(52) = 4.57, *p* < .001. CCD participants also reported most Domain scores significantly below those of the Australian normative sample (*p* ≤ .01). The only exceptions were for Standard of Living (*M* = 74.5) and Safety (*M* = 77.2). Given the low precision of the estimates indicated by *p*-values of 0.217 and 0.506 respectively, it is not possible to detect a difference for those domains.

**Table 2:** PWI means for sample of Australian adults with CCD (*N* = 53) compared with Australian population norms from studies 2002–2021 (*N* = 36) with > 65,000 participants [46]

	Adults with CCD (*N* = 53 participants)			Australian population norms Studies 2002–2021 (*N* = 36 surveys) (> 65,000 participants)				
PWI Domain	Mean	SD	CI 95%	Mean	SD	CI 95%	t	*P*-value
Whole of Life	66.6	18.9	(61.39, 71.82)	77.4	0.9	(77.10, 77.70)	4.15	0.000
PWI	65.6	15.8	(61.23, 69.93)	75.5	0.7	(75.26, 75.74)	4.57	0.000
Standard of Living	74.5	21.4	(68.64, 80.41)	78.2	1.2	(77.79, 78.61)	1.25	0.217
Health	64.0	18.4	(58.88, 69.04)	74.2	0.9	(73.90, 74.50)	4.04	0.000
Achieving in life	61.9	21.0	(56.11, 67.66)	72.1	1.2	(71.69, 72.51)	3.54	0.001
Relationships	60.6	26.1	(53.37, 67.77)	78.2	1.3	(77.66, 78.54)	4.88	0.000
Safety	77.2	22.0	(71.12, 83.22)	79.2	1.9	(78.56, 79.84)	0.67	0.506
Community	61.3	25.1	(54.40, 68.24)	70.1	1.0	(69.76, 70.44)	2.54	0.014
Future Security	59.6	25.6	(52.55, 66.69)	68.7	1.5	(68.19, 68.21)	2.57	0.013

**Fig. 2:**
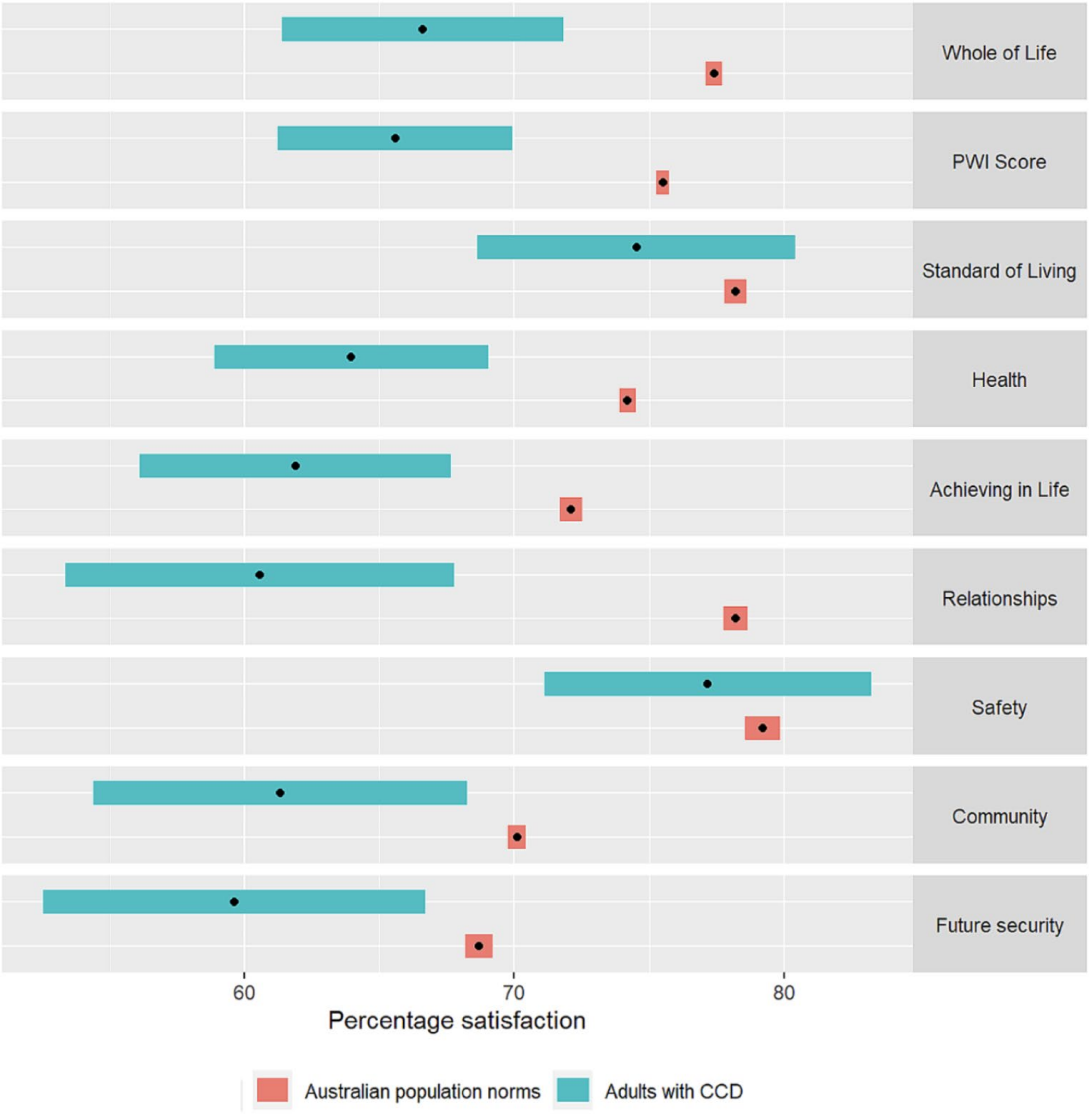
Comparison of personal wellbeing index of adults with CCD with Australian population norms. Results were plotted, with green bars indicating the 95% confidence interval (CI) for the responses of adults with CCD. Orange bars indicate the normative ranges for the general Australian population, depicted as a 95% CI of the mean responses over 36 surveys from the years 2002–2021 [46]. All point estimates of means are represented as black dots

Figure 2 shows the means and distributions (95% CI) for the CCD respondents in comparison to those for the Australian normative population for Life as a Whole, the PWI Score, and each of the seven Domain scores. It is evident that the CCD participants are, overall, responding with ratings below what might be expected of the adult Australian population, reiterating results presented in Table 2. Furthermore, there is evidence of greater variability across the CCD respondents when compared to the Australian adult population, possibly due to the much smaller sample.

### Qualitative results

To further understand the factors influencing the statistical results, we examined the qualitative free text responses provided at the end of the survey. Open-ended responses were provided by 48 of the 53 (91%) participants. Using the PWI Domain constructs as a framework, deductive thematic analysis identified issues relating to the PWI Domains. More than half described challenges relating to Achieving in Life, Personal Relationships, and Life as a Whole. Difficulties in the domains of Community Connectedness and Health were each mentioned by 40% of participants, and Future Security by almost one third of participants. Only two participants commented on Safety and no comments related to Standard of Living. Participants reported diverse impacts affecting Life as a Whole, emphasizing CCD heterogeneity and hidden disability, which were not well supported or understood by others; for example, *“I get frustrated with having to constantly explain it to GPs [General Practitioners]”* (P32). Key findings are further reported below.

### Achieving in life

Participants indicated that negative impacts of CCDs, for example, deficits in learning and memory, reduced cognitive processing speed, difficulties completing complex tasks, and anxiety, created barriers to their capacity to achieve in life. One participant reported “*forgetting where I place items or if I have showered, had a meal or even slept*” (P16). Additionally, problematic relationships with friends, family and employers and experiences of exclusion and loneliness were reported. Some described how it was difficult to feel that achievements were possible because of the impacts of societal challenges:

*I would have to say broadly, for me, the greatest difficulty is keeping step with a world made for high functioning people. It’s not just the bells and whistles of modernity, the pace of life, it’s also having the ‘spoons’ and the right culture to develop quality relationships! (P11)*

*I never seem to fit in. I feel very lost. I was called a spastic in inverted commas at school and never fitted in. At this time, I have no friends and no real life. (P44)*

Although demographic data indicated that a quarter had achieved a graduate or post graduate university degree and almost half were in full time or part time paid employment, only a few participants described positive achievements in the narrative responses, citing pride in academic achievements and raising a family.

### Personal relationships and community connections

Multiple difficulties relating to Personal Relationships were reported, with only one participant reporting *“a happy and safe life”* (P10). Many participants expressed a desire for relationships but described experiencing loneliness, social anxiety, and difficulty making and maintaining friendships:

*It* [CCD] *has a big impact on how we interact with the people around us and how complex social situations really are. Without a corpus callosum I’ve struggled to make friends but once I have a few good friendships, I find them difficult to maintain in the long run, without feeling burned out or unsure if they are making a positive impact on my life. (P33)*

Factors described as making relationships more challenging for participants included difficulties with social skills and the hidden nature of their disability. For example, one participant described how having a hidden disability affected relating to others:

*I find it extremely hard to connect to people. I find it hard to make and keep friendships and relationships. I have many issues and challenges, but people think I am okay because they can’t see anything wrong. (P25)*

Others experienced being perceived as different, causing exclusion: *“I would just like to be accepted as a normal person. People look at me differently”* (P47). *“My CCD impacts on my ability to fit in at work, and I often feel like a misfit in my own family”* (P48). For some, childhood bullying continued as family violence in adulthood: *“I was bullied during my high school days and also verbal and other abuse in my younger years, with family violence, and right through my adult life”* (P5).

### Mental and physical health

Health related issues described mental and physical health problems. Anxiety and mood disorders were reported, in addition to physical problems related to weight, coordination, hygiene, and sleep. Conditions associated with CCD, such as epilepsy, chronic pain, and hypotonia were reported as compounding impacts. Responses largely described being excluded, unsupported, lost in the system, and “*exasperated by a sick society*” (P11). In contrast one participant described CCDs as a broad spectrum, offering a perspective in which a CCD could have minimal impact on capacity to function effectively in society.

*I’m also an introvert, but at the same time I’m comfortable delivering lectures and presentations to potentially large crowds of people, often using two languages … I think the point needs to be made regularly that there is wide variation amongst those with CCD diagnoses and at one end of the spectrum are those like me for whom it has not had much of an impact. (P50)*

### Future security

Future Security findings emphasised the need for greater knowledge and awareness of CCD. Reports ranged from multiple expressions of frustration around lack of autonomy, unachievable expectations, misdirected societal perceptions, and misunderstood impacts, to a few reports of a CCD having minimal effects. One participant described reprioritising expectations of life to create a pathway to positive SWB:

*Previously my self-assessment would have been significantly more negative however, my CCD and related diagnoses have forced me to reprioritise my life and be more content with less, and to shift my expectations of what a good life means to me (P52).*

## Discussion

This is the first study to examine the SWB of people with a CCD. It provides the first evidence of reduced SWB for Australian adults with a CCD when compared with the general population and poses questions for future research and service developments.

The satisfaction scores for PWI, Life as a Whole, and five of the seven PWI Domains were significantly reduced when compared with Australian norms. Qualitative analysis of text responses provided insight into respondents’ self-perceptions of well-being, with reports of adverse life circumstances, diverse functional and social challenges, and mental health issues. It is possible that educational, vocational and social barriers have affected capacity and experiences of anxiety and depression have challenged participants, putting some at risk of chronic homeostatic failure. Results for two domains (Standard of Living and Safety) were not found to be statistically different from the Australian norms. Subsequently, it was speculated that this might be accounted for given approximately 80% were living with a spouse, partner or within the family unit, reflecting a good standard of living in a safe environment. However, further investigation in a future study is warranted.

### Maintaining stable SWB

A normally functioning homeostatic SWB system sits at or above 70 PWI points, typically fluctuating around a point of 75.5 [40]. More than half (51%) of the participants in our study recorded a PWI score below 70 points, which indicates challenged homeostasis. Furthermore, 23% of participants scored at or below 50 PWI points, which indicates risk of homeostatic failure and depression [57]. Such scores are rare in higher income countries, occurring in only 4.4% of the Australian population [18]. Although a rare condition does not necessarily cause homeostatic failure, impacts of disability that persistently challenge SWB can reduce life satisfaction to the point of defeated homeostatic control [18, 44]. In the broader literature, Cummins [40] describes the ‘Golden Triangle’, a triumvirate known to defend against homeostatic failure and affect SWB levels. The three key components comprise personal relationships, financial control, and active engagement to provide a sense of purpose. [40, 43, 58]. Challenges related to each of these components were evident for those with CCD.

The most significant difference in mean PWI Domain scores between study participants and Australian norms occurred for Personal Relationships (17.6 pp). PWI scores were supported by narrative, with participants reporting that having a CCD created challenges relating to acceptance, loneliness, stigma and abuse, affecting personal relationships and mental health and contributing to exclusion and isolation in the community. Such challenges to relationships are supported by CCD literature reporting a range of impairments affecting psychosocial and executive functioning skills for adults with CCD [14, 15, 17, 60–62]. Additionally, conducted since 1938, the longitudinal Study of Adult Development at Harvard Medical School [63], has identified ‘relationships’ as the most important factor in predicting well-being. Good relationships promote good mental and physical health. They keep us happier, healthier, and living longer. Loneliness can be toxic. It affects health (particularly cardiovascular function), shortens life and causes decline in brain function [63]. It is evident that isolation and exclusion are contributing to reduced psychosocial capacity in adults with CCD.

Participants reported reduced satisfaction with their lives as a whole, what they were achieving in life and a having a secure future, with Future Security returning the lowest mean score (59.6 pp). PWI results and participant text responses indicated that misunderstood and under-supported CCD impacts had compromised community connection, affecting education and learning, access to employment, and capacity to create a secure future. Financial control can contribute to feelings of future security providing the flexibility to meet challenges in daily life. However, for people with disability, many factors undermine financial control and consequently SWB, including discrimination and reduced access to employment. Only 53% of working age Australians with disability are in the labour force, compared with 84% without disability [44, 64]. Reflecting this population norm, fewer than half the participants (47%) in this study were engaged in full or part time employment. Almost half (45%) reported Centrelink (government pensions) as their primary income source and also recorded the lowest PWI mean score (m = 61.5 pp). A sense of achievement appears more likely where participants have gained a higher education degree and are employed. Unemployment undermines SWB in many ways, including reduced financial stability and reduced social connectivity, but further research is needed to examine possible connections within this cohort.

The results have identified key issues that clearly indicate significant concerns for those with CCD. However, the Australian policy space is complex in terms of providing support. We need further research to better understand the factors affecting SWB for this cohort and how best to inform policy and practice to ultimately improve their personal well-being.

### Strengths and limitations

Although data collection occurred during the COVID-19 pandemic, correlating national PWI surveys conducted in a similar period, from 2020 to 2022 (*N* = 4000), reported continued stability and resilience in national SWB, with PWI scores in the normative range [65]. Furthermore, very little CCD research has included participants with intellectual disability [15]. COVID-19 lockdowns confined this study to online data collection, further excluding participants with intellectual disability and those with limited technological skills and access, in addition to creating possible quality control issues around independent participation. Although one quarter of participants had completed university degrees and almost half were in paid employment, further research is needed to examine possible relationships between these data and SWB. It is important that future research provides inclusive participatory approaches and opportunities for all adults with CCD.

## Conclusion

Compared to the Australian population, adults with a CCD report significantly lower Whole of Life and Personal Wellbeing satisfaction, particularly in the Domains of Personal Relationships, Achievement in Life and Future Security. Overall, their PWI results are indicative of a defeated homeostatic state and suggest elevated vulnerability to poor mental health. The qualitative data accompanying the quantitative PWI results provide important insights into the areas of support that might make a positive difference. Some of these areas include addressing stigma and low expectations in the wider community, loneliness, social anxiety, difficulty making and maintaining friendships, together with access to education and employment. Further in-depth research is needed to gain greater insight into why SWB is significantly reduced for this cohort. Going forward, we propose employing a participatory research approach that would assist with developing a deeper understanding of the issues affecting those with CCD, improve self-advocacy and better inform policy and practice.

## Data Availability

For data access please refer to corresponding author.
